# Whole transcriptome sequencing of the aging rat brain reveals dynamic RNA changes in the dark matter of the genome

**DOI:** 10.1007/s11357-012-9410-1

**Published:** 2012-05-04

**Authors:** Shona H. Wood, Thomas Craig, Yang Li, Brian Merry, João Pedro de Magalhães

**Affiliations:** 1 Integrative Genomics of Ageing Group, Institute of Integrative Biology, University of Liverpool, Liverpool, UK; 2 The Wellcome Trust Centre for Human Genetics & MRC Functional Genomics Unit, University of Oxford, Oxford, UK

**Keywords:** RNA-seq, Cerebral cortex, Aging, Non-coding RNA

## Abstract

Brain aging frequently underlies cognitive decline and is a major risk factor for neurodegenerative conditions. The exact molecular mechanisms underlying brain aging, however, remain unknown. Whole transcriptome sequencing provides unparalleled depth and sensitivity in gene expression profiling. It also allows non-coding RNA and splice variant detection/comparison across phenotypes. Using RNA-seq to sequence the cerebral cortex transcriptome in 6-, 12- and 28-month-old rats, age-related changes were studied. Protein-coding genes related to MHC II presentation and serotonin biosynthesis were differentially expressed (DE) in aging. Relative to protein-coding genes, more non-coding genes were DE over the three age-groups. RNA-seq quantifies not only levels of whole genes but also of their individual transcripts. Over the three age-groups, 136 transcripts were DE, 37 of which were so-called dark matter transcripts that do not map to known exons. Fourteen of these transcripts were identified as novel putative long non-coding RNAs. Evidence of isoform switching and changes in usage were found. Promoter and coding sequence usage were also altered, hinting of possible changes to mitochondrial transport within neurons. Therefore, in addition to changes in the expression of protein-coding genes, changes in transcript expression, isoform usage, and non-coding RNAs occur with age. This study demonstrates dynamic changes in RNA with age at various genomic levels, which may reflect changes in regulation of transcriptional networks and provides non-coding RNA gene candidates for further studies.

**Electronic supplementary material:**

The online version of this article (doi:10.1007/s11357-012-9410-1) contains supplementary material, which is available to authorized users.

## Introduction

Brain aging frequently underlies cognitive decline and is a major risk factor for neurodegenerative conditions such as Alzheimer's and Parkinson's disease. Mental health is also a major concern of aging adults. The exact molecular mechanisms underlying brain aging, however, remain unknown (Lu et al. 2004). Quantitative analysis of aging can provide important insights into the basic mechanisms and their interactions with age-related diseases (Kirkwood 2008). Sophisticated high-throughput approaches enable age-related molecular changes to be quantified with increasing detail and may help unravel the mechanisms of aging (de Magalhães et al. 2010).

Microarrays interrogate thousands of transcripts in a cost-effective manner and have provided key insights into a number of processes, including aging. A number of such studies have been carried out in mammals with mice, rats, and humans (Lu et al. 2004; de Magalhães et al. 2009). Age-related changes in key pathways and processes, such as inflammatory and mitochondrial processes, have been identified using the microarray approach (Zahn et al. 2007; de Magalhães et al. 2009). In spite of their usefulness, microarrays have important limitations. One intrinsic problem of microarrays is their lack of sensitivity to low abundance transcripts. Elucidating the transcriptional features of aging at a more sensitive level, therefore, remains a critical challenge, but one with potential to provide mechanistic clues about aging. Microarrays also have limitations in profiling the emerging RNA complexity such as different transcripts originating from a single gene (splice variants) and non-coding transcripts (Marioni et al. 2008; Cloonan et al. 2008). This issue is particularly important for the brain because of the high complexity of RNA populations, most of which are low prevalence, as shown in a detailed comparison of Solexa/Illumina deep sequencing with conventional microarray ('t Hoen et al. 2008).

Next-generation sequencing technologies, such as the SOLiD platform from Applied Biosystems (ABI), allow large-scale sequencing at a low cost and are now driving molecular biology research (Mardis 2008; de Magalhães et al. 2010). By deeply sequencing the transcriptome and determining the frequency of each gene in the sequence sample by matching it to the genome sequence (a.k.a. RNA-Seq), one can obtain a digital measure of the presence and levels of known and unknown genes (Wang et al. 2009). Previous studies have shown that RNA-Seq is highly reproducible and has a much greater dynamic range than microarray (Cloonan et al. 2008; Mortazavi et al. 2008). RNA-Seq is also considerably more sensitive than traditional microarrays and can detect splice variants and non-coding RNAs that would otherwise go undetected (Wang et al. 2008; Tollervey et al. 2011). The capacity of the SOLiD system to survey transcriptomes in a near-complete fashion has been demonstrated in mouse embryonic stem cells by showing detection of ~50 % more genes than microarrays (Cloonan et al. 2008).

The aim of this study was to use RNA-seq to characterize the aging transcriptome in the rat cerebral cortex. It was envisaged that the comparable gross structure of the rat to the human brain would make these results potentially applicable to human brain aging and neurodegeneration research. Furthermore, the rat has been used extensively in neurological research for many years and is held as a model for mammalian behavioural and neurodegeneration studies (Jacob 1999). By profiling the transcriptome, it was hoped to gain insight into the RNA complexity of aging and the regulatory pathways involved.

This study demonstrated that in addition to changes in the expression of protein-coding genes with age, substantial changes in transcript expression and isoform usage occur. Moreover, changes in non-coding RNA expression are prevalent, suggesting that these may be of importance in brain aging. These data showcase how transcriptional changes occur at multiple genomic levels, including in the dark matter of the genome.

## Materials and methods

### Animals

A previous experiment supplied the rat brain tissue for this study (Merry et al. 2008). All animal husbandry procedures undertaken in this study were carried out in accordance with the provisions of the United Kingdom Animals (Scientific Procedures) Act 1986. Male BN rats (SubstrainBN/SsNOlaHSD) were obtained from Harlan UK at 21–28 days of age and maintained under barrier conditions on a 12-h light: 12-h dark cycle (08:00–20:00). The health status of the rats was monitored at regular intervals through the screening of sentinel animals. All rats were fed ad libitum and sacrificed at 6, 12, and 28 months of age. None of the animals exhibited any signs of pathology when sacrificed. Each age group had six rats from which brain samples were taken, flash frozen, and stored at −80°C.

### Cortex dissection and RNA extraction

To minimise thawing and therefore degradation of RNA, the cerebral cortex was removed from the whole brain on a solid CO_2_ base under a dissecting microscope. The cerebral cortex was cut into small pieces to aid RNA extraction.

RNA was extracted from the cerebral cortex using Qiagen's TissueLyser II and RNeasy lipid tissue kit. The quality of the extracted RNA was assessed using the Agilent 2100 Bioanalyser; all RNA integrity numbers (RINs) were above 8, indicating that good quality RNA had been extracted. The samples were pooled 2 by 2 (leaving 3 samples per age group). Ribosomal RNA was removed from the pooled samples using a Eukaryote ribominus kit (Invitrogen) and confirmed with the Agilent 2100 Bioanalyser. Ribosomal removal, rather than Poly-A selection, allows certain non-coding RNAs without Poly-A tails to be included in the sequencing.

### cDNA library preparation and SOLiD sequencing

The library preparation protocol was carried out according to the manufacturer's instructions: The RNA was fragmented and cleaned up using spin columns (Invitrogen) and SOLiD RNA adapters were then hybridized and ligated to the samples. Reverse transcription was performed to generate cDNA. The cDNA was purified, size selected, amplified, and then purified again (as detailed in the SOLiD protocol). The size distribution of the cDNA library was assessed using the Bioanalyser. The samples were then subject to emulsion PCR and sequenced in the Centre for Genomic Research at the University of Liverpool using the SOLiD system V4, in the forward and reverse.

### Mapping

The RNA-seq results from the SOLiD system were output as color space FASTA and quality files, these were converted into FASTQ format using a python script from Galaxy (http://main.g2.bx.psu.edu/). The FASTQ files were mapped to the Ensembl release 65 rat reference genome (RGSC 3.4 assembly, May 2010 gene build) using Bowtie (Langmead et al. 2009) and settings appropriate to SOLiD data. For each sample, approximately 33.6 million reads were generated (range, 29.5 to 39.8 million reads). On average 16.7 million reads per sample were mapped to the reference genome (range, 13.8 to 21.4 million reads, approximately 50 % of reads generated were mapped). All data have been submitted to GEO under the accession GSE34272.

### Gene expression analysis

In order to measure gene expression from mapped data, the BAM files from Bowtie mapping were sorted using SAMtools (Li et al. 2009). Raw counts per gene were estimated by the Python script HTSeq count (http://www-huber.embl.de/users/anders/HTSeq/) using the Ensembl rat reference genome. The raw counts per gene were used by EdgeR (Robinson and Oshlack 2010) to estimate differential expression (DE).

EdgeR (Bioconductor release 2.9) uses a pair-wise design to measure differential expression. The analysis is based on a negative binomial model that uses over-dispersion estimates to account for biological variability (i.e., sample to sample differences); this is an alternative to the Poisson estimates of biological variability that are often inappropriate (Oshlack et al. 2010). Genes with less than 5 reads were excluded from the analysis and TMM normalisation of the sequenced libraries was performed to remove effects due to differences in library size (Robinson and Oshlack 2010). The most stringent dispersion method (tag-wise) was used to ensure that differential expression was not due to individual differences (EdgeR tag-wise options: prior.*n* = 7, prop.used = 0.5, gridlength = 500). EdgeR generates a fold change for each gene, *p* values and the Benjamini-Hochberg false discovery rate (FDR) are calculated to statistically test the measured DE. As in previous studies, no effect size cut-off was set, as aging-related changes often tend to be subtle (de Magalhães et al. 2009).

### Splice variant prediction and expression analysis

In order to predict splice variant usage with age, the sequence data must be mapped using Tophat (Trapnell et al. 2009), which uses Bowtie initially (as previously) but then generates splice variant predictions. The Cufflinks pipeline (Trapnell et al. 2010) (cufflinks, cuffcompare, cuffdiff) was used to assemble the transcripts (known and novel), assess the usage of splice variants, promoters, and coding sequences.

Cuffdiff can be used to measure the DE of transcripts. It has been reported, however, that the DE FPKM (fragments (reads) per kilobase of exon model per million) methods used in these analyses do not account for biological variation and can lead to false positive results (Oshlack et al. 2010). To gain the most robust results for transcript DE, raw counts were calculated for each transcript with HTSeq using the combined GTF file generated by Cufflinks as the genomic reference file (lists the splice variants and their genomic location generated by Tophat). Differential transcript expression was then tested with EdgeR, as in gene DE, making the results more robust and comparable to the gene expression results.

Cuffdiff also predicts splice variant, promoter, and coding sequence usage changes (a.k.a. overloading). Overloading is measured in each group and computed from the relative abundance of transcripts (measured by the expected fragments per kilobase of transcript per million fragments mapped, a.k.a FPKM). The square root of the Jensen-Shannon divergence computed on the relative abundances of the sequences is the test statistic from which a *p* value is generated. The *p* value is corrected using the Benjamini-Hochberg correction for multiple-testing giving a FDR.

### qPCR

To generate cDNA for qPCR, 3.5 μg of total RNA was reverse transcribed using Superscript III First-strand synthesis system for RT-PCR (Invitrogen, Paisley, UK). The Roche universal probe library designer was used to design primers (https://www.roche-applied-science.com/servlet/) with sequences obtained from Ensembl. All primers were designed to cross an exon–exon boundary. The specificity of the primers was checked using BLAST (http://www.ncbi.nlm.nih.gov/BLAST/). A reference gene experiment was conducted to identify the most stably expressed genes in the cerebral cortex with age (data not shown); HPRT1 (supplementary Table 2) and YWHAZ (Rn00755072_m1 Applied Biosystems) were the most stably expressed with age and were used to normalise the qPCR results. Supplementary Table 1 shows the primers, amplicons, and probes used.

The qPCR assays were all performed in triplicate using a TaqMan™ ABI PRISM 7500 fast (Applied Biosystems, Foster City, CA, USA) in 96-well plate format. A 20-ml reaction volume was used per well, consisting of: 10 μl Taqman 2× PCR master mix (Universal PCR Mastermix; Applied Biosystems), 0.2 μl each of 20 mM forward and reverse primers, 0.2 μl of 10 mM probe (Exiqon; Roche Diagnostics Ltd.), 0.2 μl distilled water and 9.2 μl of cDNA or water for the negative controls. The amplification was performed as follows: 2 min at 50°C, 10 min at 95°C followed by 40 cycles of 95°C for 15 s and 60°C for 1 min. The efficiency of the assays were between 93 % and 107 % and the *R* ^2^ values were >0.98. The ΔΔcT method was used to measure expression; “6 months” was used as the reference samples from which relative expression was calculated in 12- and 28-month-old rats. The data were further corrected by the efficiency of the standard curve for each gene. Log2 fold change relative to “6 months” was calculated and compared to the RNA-seq results in order to confirm the expression results. The standard error was calculated for log2 fold change as follows: (std error/mean)*log2e. For qPCR the relative quantification values (calculated from 6 month—sample 1) were used to calculate standard error. For RNA-seq, raw reads converted into relative values (calculated from 6 month—sample 1) were used to calculate standard errors.

### Novel non-coding RNA

Novel non-coding RNAs (ncRNA) were identified by Cufflinks and shown to be DE with age. To identify the types of ncRNA DE, they were split by size into the following groups: <200nt and >200nt. <200nt non-coding RNA was checked using a combination of miRbase (Kozomara and Griffiths-Jones 2011), RFAM (Gardner et al. 2011), and RNAFold (Hofacker 2003) to identify miRNA, snoRNA, or other RNA.

Transcripts (>200nt) were thought to be long non-coding RNAs (lncRNAs). To test this, transcripts were checked for conserved protein domains using EMBOSS transeq (http://www.ebi.ac.uk/Tools/emboss/transeq) and NCBI's CDD-search tool (in all six reading frames) (Marchler-Bauer et al. 2011). Any transcript with a conserved protein domain was not defined as an lncRNA. For each of the novel lncRNA, the top ten closest protein-coding genes (in terms of genomic location) were obtained from Ensembl, in order to test co-localisation of expression with our genes DE with age (Ponjavic et al. 2009).

## Results

RNA was obtained from the cerebral cortex of 6-month-, 12-month-, and 28-month-old rats. The SOLiD platform was used for whole transcriptome sequencing at a high coverage and reads were mapped to the reference rat genome (see “Materials and methods”). Reads per gene represent a quantitative digital measure of expression levels. If a gene failed to pass our selection threshold across all samples, it was defined as not expressed, leaving 16,152 protein-coding genes and 2,491 non-coding genes that were expressed in at least one cerebral cortex sample. In order to assess gene expression changes with age, pairwise comparisons of 6- vs. 12-month-olds, 6- vs. 28-month-olds, and 12- vs. 28-month-olds were tested for differential expression (DE) using a method that controls for false discovery rates (see “Materials and methods”). Changes in gene expression are stated as the log2-transformed fold change (log2FC) in expression with increasing age.

### Gene expression changes

**Fig. 1:**
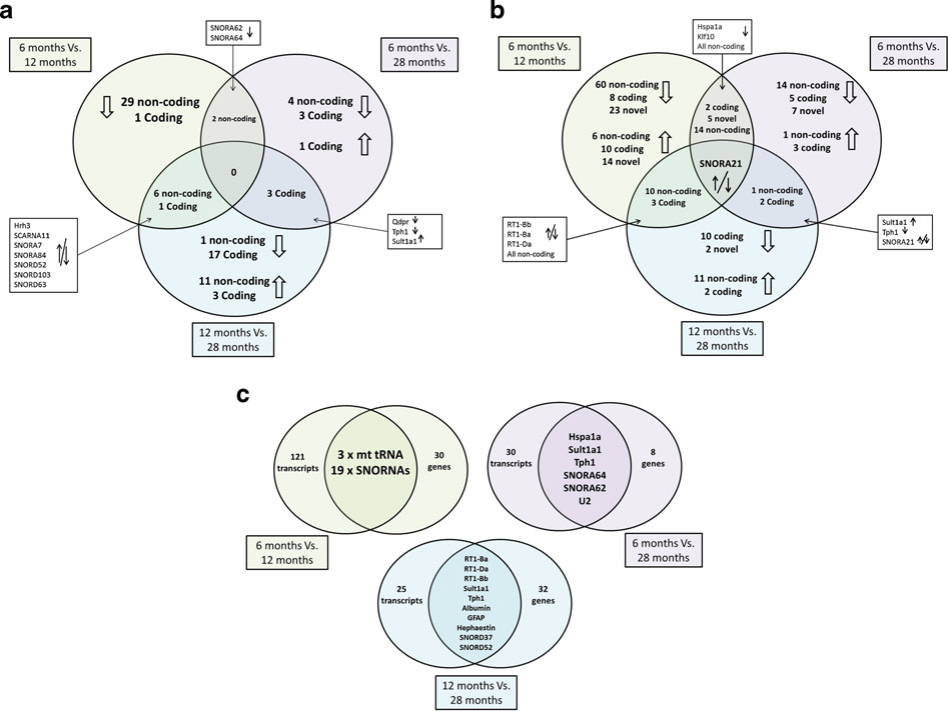
Venn diagrams for differentially expressed genes and transcripts (FDR <0.05). **a** Differentially expressed genes in 6- vs. 12-month-old, 6- vs. 28-month-old, and 12- vs. 28-month-old rats. *Arrows* indicate the direction of fold change with increasing age. Both *up* and *down arrows* indicate a quadratic change with age. Genes differentially expressed in more than one age group comparison are listed. **b** Differentially expressed transcripts in 6- vs. 12-month-old, 6- vs. 28-month-old, and 12- vs. 28-month-old rats. *Arrows* indicate the direction of fold change with increasing age. Both *up* and *down arrows* indicate a quadratic change with age. Protein coding transcripts differentially expressed in more than one age group comparison are listed. **c** The overlap between differentially expressed transcripts and genes at all ages, 6- vs. 12-month-old, 6- vs. 28-month-old, and 12-vs. 28-month-old rats

Figure 1a summarises the DE genes for all age comparisons (FDR < 0.05). Table 1 lists all protein coding genes differentially expressed in all pairwise age comparisons. When comparing 6- to 12-month-old rats, the majority of DE genes were non-coding (see Fig. 1a and supplementary Table 2).

**Table 1:** Protein-coding genes significantly differentially expressed at 6- to 12-, 6- to 28-, and 12- to 28-month-old rats

Ensembl ID	Gene symbol	Gene name	Log2 fold change	FDR
6- vs. 12-month-old rats				
ENSRNOG00000008080	Hrh3	Histamine H3 receptor	−2.02	0.002
6- vs. 28-month-old rats				
ENSRNOG00000019342	Sult1a1	Sulfotransferase 1A1	1.74	3.16 × 10^−07^
ENSRNOG00000033526	Hspa1a	Heat shock 70 kDa protein 1A/1B	−2.11	0.021
ENSRNOG00000011672	Tph1	Tryptophan 5-hydroxylase 1	NE 28	0.022
ENSRNOG00000003253	Qdpr	Dihydropteridine reductase	−0.94	0.033
12- vs. 28-month-old rats				
ENSRNOG00000000451	RT1-Ba	Rano class II histocompatibility antigen, B alpha	−1.82	8.40 × 10^−11^
ENSRNOG00000033215	RT1-Db1	Rano class II histocompatibility antigen, D-1 beta	−2.14	8.40 × 10^−11^
ENSRNOG00000032844	RT1-Da	RT1 class II, locus Da	−1.86	5.78 × 10^−09^
ENSRNOG00000018735	Cd74	H-2 class II histocompatibility antigen gamma	−1.69	2.00 × 10^−07^
ENSRNOG00000019342	Sult1a1	Sulfotransferase 1A1	1.54	2.91 × 10^−07^
ENSRNOG00000002919	Gfap	Glial fibrillary acidic protein	−1.29	5.89 × 10^−07^
ENSRNOG00000032708	RT1-Bb	Rano class II histocompatibility antigen, B-1 beta	−1.83	2.44 × 10^−05^
ENSRNOG00000002911	Alb	Serum albumin	−2.19	8.80 × 10^−05^
ENSRNOG00000005542	Apob	Apolipoprotein B-100Apolipoprotein B-48	−2.60	3.06 × 10^−04^
ENSRNOG00000011672	Tph1	Tryptophan 5-hydroxylase 1	NE 28	4.36 × 10^−04^
ENSRNOG00000030729	E9PSV0_RAT	Complement component 4, gene 2	−1.78	0.004
ENSRNOG00000031230	LOC689064	Hemoglobin subunit beta-2	−1.14	0.004
ENSRNOG00000030625	Tf	Signal recognition particle receptor subunit beta	−0.92	0.005
ENSRNOG00000012294	Heph	Hephaestin	1.11	0.007
ENSRNOG00000003253	Qdpr	Dihydropteridine reductase	−0.85	0.010
ENSRNOG00000000853	Aif1	Allograft inflammatory factor 1	−1.37	0.015
ENSRNOG00000008080	Hrh3	Histamine H3 receptor	1.20	0.031
ENSRNOG00000007227	RGD1306682	Similar to RIKEN cDNA 1810046 J19 (RGD1306682), mRNA	−0.93	0.039
ENSRNOG00000000574	F1LTG5_RAT	Uncharacterized protein	−1.13	0.042
ENSRNOG00000009263	Ifi27	Interferon, alpha-inducible protein 27	−1.11	0.042

NE—not expressed (reads below 5 in all samples), *6* = 6-month-old, *12* = 12-month-old, and *28* = 28-month-old. FDR < 0.05 (fold change represents change with increasing age)

Between 6- and 28-month-old the protein-coding genes Qdpr, Tph1, Sult1a1, and Hspa1a were DE, with Qdpr, Tph1, and Sult1a1 showing a continuation of the trend at 12 vs. 28 months. SNORA62 and SNORA64 also show DE in two age comparisons suggesting that these genes are up-regulated at early age and then down-regulated with increasing age (see Fig. 1a and supplementary Table 2).

Between 12- and 28-month-olds, the majority of protein coding genes are down-regulated and non-coding are up-regulated. Functional enrichment analysis of DE protein-coding genes from 12- to 28- month-olds using DAVID (Huang et al. 2009) revealed that “Antigen processing and presentation via MHC class II” was significantly over-represented in the DE genes (Enrichment 3.87, FDR 6.5 × 10^−7^). This is due to a down-regulation of genes from the MHC class II family, e.g., RT1-Da, CD74, and other immune related genes; complement component 4-gene 2, Ifi27, Aif1. As in the 6 to 12 month comparison, Hrh3 and six snoRNAs are significantly DE. Their expression profile, however, is inverted, suggesting a quadratic change in expression over the three ages (see Fig. 1a, Table 1 and supplementary Table 2).

### qPCR confirmation of observed expression

**Fig. 2:**
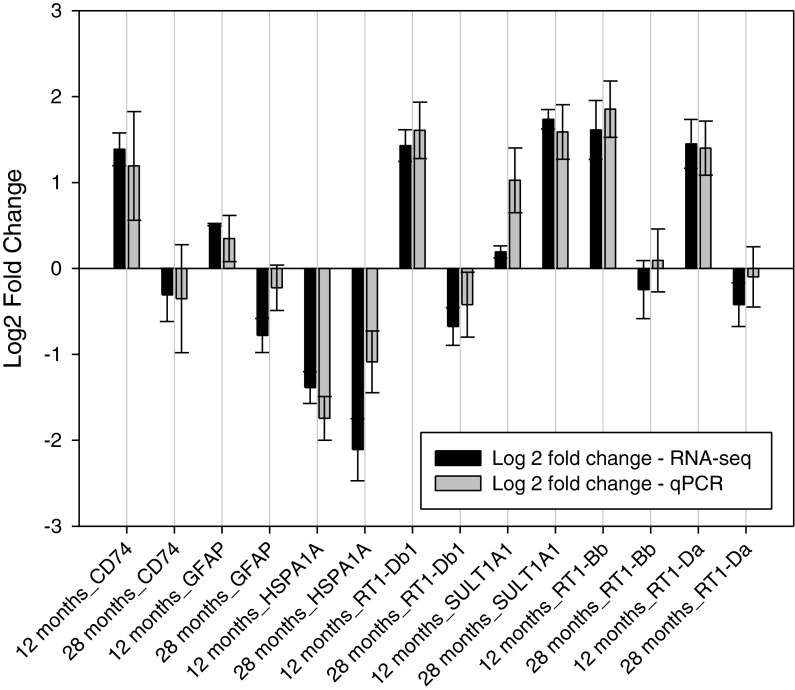
qPCR confirmation of observed expression. The expression levels (log2 fold change) of seven genes relative to 6-month-old rats are displayed for RNA-seq and qPCR in 12- and 28-month-old groups. The *error bars* are the standard error calculated for log2 fold change as follows: (std error/mean)*log2e. For qPCR, the relative quantification values (calculated from 6-month-old—sample 1) were used to calculate standard error. For RNA-seq, raw reads converted into relative values (calculated from 6-month-old—sample 1) were used to calculate standard error

**Fig. 3:**
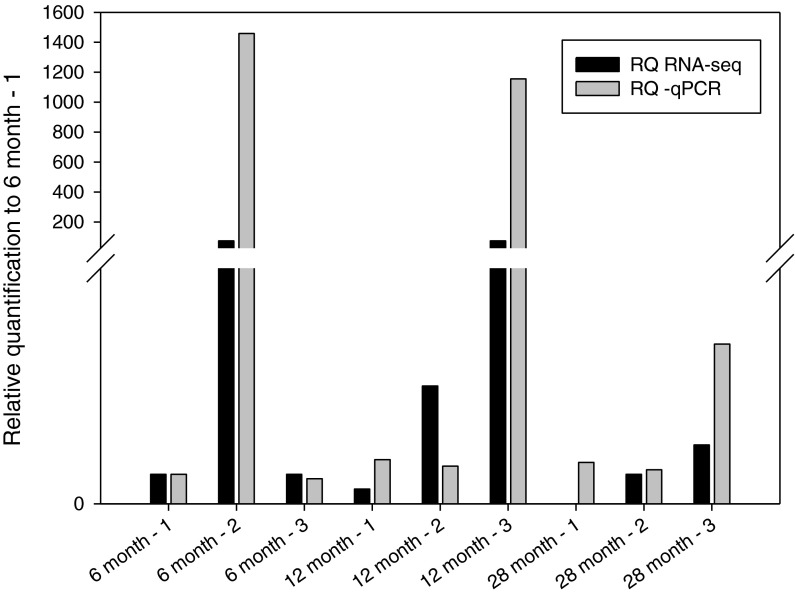
Tph1 expression relative to 6-month sample 1 using qPCR and RNA-seq. This graph shows the relative quantification values (calculated from 6-month-old—sample 1) for each sample for qPCR and RNA-seq (raw reads were used)

Eight genes, with a range of significance values, were selected for confirmation by qPCR: CD74, GFAP, Hsp1a1, RT1-Db1, Sult1a1, Tph1, RT1-Bb, RT1-Da (primer and probe information is in supplementary Table 1). Figure 2 shows the log2FC from qPCR and RNA-seq. Seven genes confirm the expression profile observed in the RNA-seq experiment (Tph1 on Fig. 3). One gene, RT1-Bb, confirmed the observed expression profile when comparing 6- to 12-month-old animals but not in the 6- to 28-month-old comparison; however, the log2FC is nominal (RNA-seq, −0.25, qPCR, 0.09).

Tph1 showed the same pattern of expression at 12- vs. 28-month-olds using qPCR and RNA-seq (qPCR −2.38 and RNA-seq −4.62, data not shown). It was noted, however, that the standard error was high, therefore, in order to confirm the RNA-seq results, the relative expression of each sample were compared. Figure 3 shows that qPCR and RNA-seq strongly agree for each individual sample confirming the observed expression in the RNA-seq experiment, despite the high standard error when the fold change for each age group is calculated. Figure 3 also shows that “6 months sample 2” and “12 months sample 3” are likely to be skewing the DE result, giving the observed significant DE between 6-/12- month-olds and 28-month-old rats. This is despite using tag-wise dispersion to minimise the effect of individual differences (see “Materials and methods”).

### Transcript expression changes with age

When quantifying gene expression, multiple transcripts from a given gene are grouped together to produce an average expression value for the gene. Large changes in an individual transcript may not be reflected in the average gene expression value. The expression of individual transcripts is biologically relevant information, as different transcripts from the same gene can produce different proteins. It is important, therefore, to assess DE for each individual transcript; RNA-seq makes this possible. Using the Tophat software, the reads are assembled into transcripts, rather than genes, as in the Bowtie program. This allows quantification at the individual transcript level and prediction of novel transcripts.

Applying the same DE methods as the gene analysis, Fig. 1b summarises DE expressed transcripts for all age comparisons. Table 2 and supplementary Table 3 list the protein-coding and non-coding transcripts DE, respectively. More transcripts were DE compared to genes, except in the 12- vs. 28-month comparison (Fig. 1c). Multiple novel transcripts were identified as DE (Fig. 1b), for classification and genomic location of these transcripts, see supplementary Table 4.

**Table 2:** Protein-coding transcripts significantly differentially expressed at 6- to 12-, 6- to 28-, and 12- to 28-month-old rats

Ensembl ID	Gene symbol	Gene name	Log2 fold change	FDR
6- vs. 12-month-old rats				
ENSRNOT00000048332	RT1-Da	Rano class II, locus Da	2.60	1.29 × 10^−11^
ENSRNOT00000000523	RT1-Ba	Rano class II histocompatibility antigen, B alpha	2.39	1.67 × 10^−11^
ENSRNOT00000000525	RT1-Bb	Rano class II histocompatibility antigen, B-1 beta	3.03	1.37 × 10^−10^
ENSRNOT00000049667	Hspa1a	Heat shock 70 kDa protein 1A/1B	−2.42	3.69 × 10^−05^
ENSRNOT00000027346	Cdh1	Cadherin-1E	2.63	2.46 × 10^−04^
ENSRNOT00000067419	Klf10	Krueppel-like factor 10	NE 12	0.002
ENSRNOT00000004174	Gc	Vitamin D-binding protein	NE 6	0.004
ENSRNOT00000058641	Egr3	Early growth response protein 3	−1.39	0.012
ENSRNOT00000051137	F1LXJ8	Uncharacterized protein	Ne12	0.013
ENSRNOT00000037445	D3ZVJ6_RAT	Uncharacterized protein	1.04	0.013
ENSRNOT00000049259	Satb2	DNA-binding protein SATB2	−1.20	0.014
ENSRNOT00000004703	Nptxr	Neuronal pentraxin receptor	−0.98	0.015
ENSRNOT00000022226	LOC100364907	Family with sequence similarity 18, member B2	1.75	0.026
ENSRNOT00000035085	Usp40	Ubiquitin carboxyl-terminal hydrolase 40	NE 6	0.028
ENSRNOT00000021181	D4A357_RAT	Uncharacterized protein	1.28	0.032
ENSRNOT00000022202	Sema3b	Semaphorin-3B	NE 6	0.034
ENSRNOT00000050716	Lrrc10b	Leucine-rich repeat-containing protein 10B	−1.86	0.035
ENSRNOT00000003013	Ptplb	Uncharacterized protein	−1.60	0.041
ENSRNOT00000054997	D3ZWX7_RAT	Uncharacterized protein	−1.01	0.042
6- vs. 28-month-old rats				
ENSRNOT00000049667	Hspa1a	Heat shock 70 kDa protein 1A/1B	−3.21	6.82 × 10^−08^
ENSRNOT00000026186	Sult1a1	Sulfotransferase 1A1	1.74	1.1 × 10^−06^
ENSRNOT00000056109	Tph1	Tryptophan 5-hydroxylase 1	NE 28	5.2 × 10^−06^
ENSRNOT00000026121	Hmgcs2	Hydroxymethylglutaryl-CoA synthase, mitochondrial	1.64	0.003
ENSRNOT00000005157	Tph2	Tryptophan 5-hydroxylase 2	NE 28	0.006
ENSRNOT00000067419	Klf10	Krueppel-like factor 10	NE 28	0.015
ENSRNOT00000067442	Arc	Activity-regulated cytoskeleton-associated protein	−1.01	0.042
ENSRNOT00000056120	RGD1359529	UPF0471 protein C1orf63 homolog	1.00	0.042
12- vs. 28-month-old rats				
ENSRNOT00000000523	RT1-Ba	Rano class II histocompatibility antigen, B alpha	−1.79	1.49 × 10^−06^
ENSRNOT00000048332	RT1-Da	Rano class II, locus Da	−1.87	1.49 × 10^−06^
ENSRNOT00000056109	Tph1	Tryptophan 5-hydroxylase 1	NE 28	2.02 × 10^−05^
ENSRNOT00000026186	Sult1a1	Sulfotransferase 1A1	1.48	1.74 × 10^−04^
ENSRNOT00000003921	Alb	Serum albumin	−2.62	4.08 × 10^−04^
ENSRNOT00000000525	RT1-Bb	Rano class II histocompatibility antigen, B-1 beta	−1.73	0.002
ENSRNOT00000034401	Gfap	Glial fibrillary acidic protein	−1.12	0.006
ENSRNOT00000047434	Fcgr3a	Fc fragment of IgG, low affinity IIIa, receptor	NE 28	0.024
ENSRNOT00000007164	Pmm1	Phosphomannomutase 1	−2.29	0.024
ENSRNOT00000015944	Trh	ProthyroliberinThyroliberin	NE 28	0.024
ENSRNOT00000050675	Lrp2	Low-density lipoprotein receptor-related protein 2	NE 28	0.040
ENSRNOT00000017312	Heph	Hephaestin	1.13	0.040

*NE*—not expressed (reads below 5 in all samples), *6* = 6-month-old, *12* = 12-month-old, and *28* = 28-month-old. FDR < 0.05 (fold change represents change with increasing age)

Figure 1c shows the overlap of transcripts and genes identified by both analyses. The 6-vs. 12-month comparison reflects the high number of non-coding genes found by the gene expression analysis. More protein-coding transcripts were DE than protein coding genes (Fig. 1b,c, Table 2). Using DAVID functional enrichment analysis on the DE protein-coding transcripts, “Antigen processing and presentation via MHC class II” (Enrichment 2.7, FDR 6.2 × 10^−4^) is significant. Cdh1, the transcription factor early growth response protein 3 and Sema3b are all DE in the 6- to 12-month-old comparison; these transcripts are all involved in development/maturation (Table 2).

Comparing 6- to 28-month-olds, protein-coding transcripts significantly DE are not in a related functional group (as assessed by DAVID); however, both Tph1 and Tph2 were DE; these are involved in serotonin biosynthesis and both were down-regulated. Hspa1a, Sult1a1, and three non-coding RNAs were also DE reflecting the gene expression results (Fig. 1c).

Between 12- and 28-month-olds, all non-coding transcripts were up-regulated and the majority of protein-coding transcripts were down-regulated (10 down and 2 up). The protein-coding transcripts showed enrichment for “Antigen processing and presentation via MHC class II” (Enrichment 2.95, FDR 1.3 × 10^−3^), as in the gene expression analysis (Fig. 1c).

The apparent discrepancy between the number of genes and transcripts identified as DE in most cases is due to genes with multiple transcripts, i.e., a large change in an individual transcript that is not reflected in the average gene expression value. However, some single transcript genes were DE in the transcript analysis only. This could be due to the transcript assembly methods used by Tophat compared to Bowtie. Tophat filters the more unreliable reads compared to Bowtie, therefore, these transcripts may be significant in the transcript expression analysis but not in the gene expression analysis (our raw reads and *p* values reflect this). All results are given in the supplements and on our website (http://genomics.senescence.info/gene_expression/RNA_seq_rat_brain.php); raw data is available in GEO (GSE34272) if others wish to redo the analysis.

### Novel RNA transcripts

Across the three pairwise comparisons, 37 novel RNA transcripts were identified as DE; these were either unknown intergenic transcripts, within introns or had multiple classifications (supplementary Tables 3 and 4). To better understand why these unknown transcripts were DE, a combination of RFAM (Gardner et al. 2011), miRbase (Kozomara and Griffiths-Jones 2011), RNAFold (Hofacker 2003), and NCBI's conserved protein domain database (Marchler-Bauer et al. 2011) were used to identify the non-coding RNA type (see “Materials and methods”). Supplementary Table 4 lists the non-coding RNA type assigned and genomic position, in brief, a novel tRNA-sec, 2 splicesomal RNAs, 10 microRNAs, 2 snoRNAs, and 14 novel putative lncRNAs were identified. Seven transcripts could not be classified by our methods either because they were smaller than the read length (possible adapter sequencing), did not show any conservation or had an unusual secondary structure.

The novel lncRNAs were tested for co-localisation with DE expressed protein-coding transcripts (Ponjavic et al. 2009). No evidence of co-localisation was found, therefore a *cis* regulatory role is unlikely. This does not, however, preclude the possibility that these novel lncRNAs are acting in *trans* or over long physical distances.

### Differential promoter, coding sequence, and splice variant usage with age

**Fig. 4:**
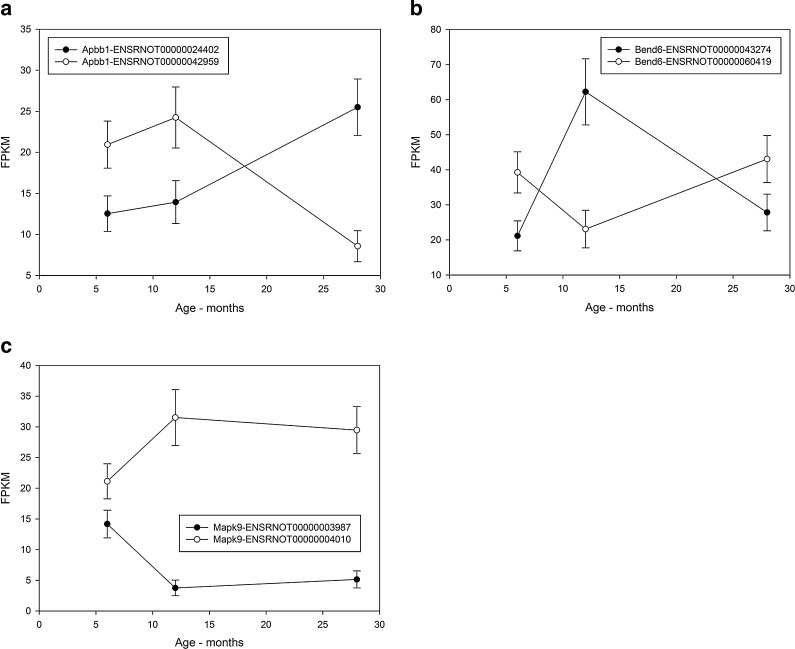
Examples of isoform switching (differential splice variant usage) at different ages. The relative abundance is given as the FPKM and the age axis is in months. *Error bars* represent the standard deviation. **a** Apbb1—Statistically significant differential splice variant usage between 6- vs. 28-month-olds and 12- vs. 28-month-olds. **b** Bend6—statistically significant differential splice variant usage between 6- vs. 12-month-olds and 12- vs. 28-month-olds. **c** Mapk9—statistically significant differential splice variant usage between 6- vs. 12-month-olds and 6- vs. 28-month-olds

For each primary transcript, it is possible to estimate the amount of overloading amongst its isoforms, i.e., differential usage of splice variants with age. Transcripts differentially spliced across the age comparisons are listed in supplementary Table 5. There is some overlap across differentially spliced transcripts between the age comparisons, which suggest that there may be isoform switching with age as in differentiation and development (Trapnell et al. 2010; Kalsotra and Cooper 2011). Figure 4 shows examples of isoform switching.

A gene can produce multiple proteins, therefore any differences in coding sequence (CDS) output with age was tested (see supplementary Table 6). A DAVID analysis revealed no significant functional clusters; however, multiple genes related to actin, cytoskeleton, and microtubule regulation/binding were present, suggesting differences in mitochondrial and other organelle transport within neurons (Sheng and Cai 2012).

Changes in promoter use, and therefore differences in the primary transcript produced with age, can be predicted by grouping splice variants by a transcription start site ID. Eleven genes show differential promoter use across the three age comparisons, seven of which show differential CDS output (see supplementary Table 6). Glutamate receptor 2 (Gria2) is the only gene with promoter use changes that shows differential splicing (Supplementary Tables 5 and 6). A DAVID analysis shows that the majority of genes with differential promoter use are “membrane bound vesicles” (using 11 genes from all age comparisons; enrichment 4.04, FDR 3.7 × 10^−3^).

## Discussion

Relative to most disease gene expression studies, few genes differentially expressed with age have been found for most organs, including the brain, when using microarrays (de Magalhães et al. 2009; Zahn et al. 2007). In spite of the greater sensitivity of RNA-seq, again relatively few genes differentially expressed with age were found in our study. Reflecting previous gene expression studies (Njemini et al. 2011; Odera et al. 2007), a heat shock protein, and a low-density lipoprotein receptor-related protein-2 were down-regulated with age (Table 1).

Development/maturation-related transcripts were identified as DE in the comparisons between 6- and 12-month-old rats. Comparing all three ages shows that some genes are expressed in a quadratic profile (inverted U- or U-shaped curve) with increasing age, e.g., Hrh3, RT1-Da, RT1-Ba, RT1-Bb, and multiple snoRNAs. A complex, non-linear pattern of maturation/age-related change has been previously observed in the human and macaque brain (Somel et al. 2010). Measures of brain aging (cognitive, sensory, motor) have also shown quadratic-like profiles, peaking at adulthood and declining with an accelerating trajectory thereafter (Fjell et al. 2010). There is also a debate of when that peak occurs and brain aging begins (20–39 years in humans) (Fjell et al. 2010). The quadratic profile observed in some genes, together with the transcript evidence at 6- vs. 12-month-olds, suggests that aging-related changes will be observed when comparing 12- to 28-month-olds and to a lesser extent 6- to 28-month-olds. These observations justify the use of pair-wise analysis instead of linear regression over all age groups.

Changes in immune system-related genes involved in antigen presentation, the complement system, and regulation of cytokines were observed in the 6- to 12-month-olds and 12- to 28-month-olds comparisons, in a quadratic-like profile. Twelve-month-old rats show an increase in the expression of MHC II genes from 6 months; this could indicate the final programming of the brain's immune system, consistent with the idea that brain maturation takes longer than other systems (greater than 20 years old in humans) (Toga et al. 2006). By 12 months, the rat the thymus has regressed, possibly resulting in the subsequent decline in immune-related genes observed between 12- to 28-month-olds. Our data shows decreases in genes belonging to the MHC II family (12- to 28-month-olds), suggesting decreased inflammation in aged cerebral cortex. This contradicts previous observations that normal aging exhibits immune activation and increased inflammation (de Magalhães et al. 2009; Zahn et al. 2007). It has been suggested, however, that detection of MHC II antigens in microglial cells (the brain antigen presenting cells) does not equal evidence of inflammation and that microglia may be neuroprotective (Graeber and Streit 2010). Further to this, it has been noted that a progressive decline of immune function occurs in other tissues with aging with naive T cells from aged animals showing decreased antigen responsiveness (Linton et al. 1996; Bruunsgaard et al. 2000). Aging may reduce the ability to respond effectively to immune challenges rather than causing inflammation in the cerebral cortex. Neurodegeneration, however, is characterised by chronic inflammation (Glass et al. 2010). An absence of serious inflammatory conditions and low levels of serum heat shock protein have been associated with successful biological aging (Njemini et al. 2011). This is demonstrated in the expression profiles observed in this study that employed disease-free, health-defined animals.

Changes in neurotransmitter levels and receptors have been noted in the aging cerebral cortex (Wong 2002). Our study identified changes in histamine receptor 3, serotonin biosynthesis enzymes Tph1, Qdpr (Table 1 and supplementary Table 1) and Tph2 (Table 2 and supplementary Table 3); however, the variability between samples for Tph1 does bring the result into question (Fig. 3). A decline in serotonin function with aging is consistent with observations of age-related changes in behaviours, such as sleep, that are linked to serotonergic function (Meltzer et al. 1998). Similarly, lipid and cholesterol metabolism/transport genes ApoB and Lrp2 were decreased in the 12- vs. 28-month-olds comparison. Both function in the recognition, transport, and clearance of LDL particles. Decreases in these genes suggest a dysfunction in lipid clearance with age, perhaps leading to protein aggregate formation (Lindner and Demarez 2009). Sult1a1 was one of the few protein-coding genes to increase in expression with age (6- to 28-, 12- to 28-month-olds). An increase of Sult1a1 has been observed in mouse hippocampus and has been associated with age-related memory deficit (Verbitsky et al. 2004).

RNA-seq allows the widespread splicing changes and alterations in the levels of various types of RNA genes to be surveyed, illustrating the usefulness of RNA-seq for studies of aging. The strong agreement of the qPCR results with the RNA-seq data demonstrates the reliability of RNA-seq, giving confidence in the observed results. Differential splicing patterns were observed with age, indicating that isoform switching, as observed for cell differentiation (Trapnell et al. 2010) is occurring during maturation (6- vs 12-month-olds) and aging (6-/12- vs 28-month-olds). The primary transcripts showing differential splice variant use are involved in a range of functions, such as translation, endocytosis, lysosome biogenesis, and kinase activity. Figure 4 shows specific examples of isoform switching in Apbb1 and MAPK9. These are involved in DNA damage response/induction of apoptosis and stress activated immune response, respectively.

Further to the changes observed in splice variant usage, promoter and CDS usage also show considerable alterations with age. The differential CDS analysis showed genes involved in mitochondrial and other organelle transport within neurons. Activity-regulated cytoskeleton associated protein (Arc) is also down-regulated between 6- and 28-month-olds (Table 2). Changes in mitochondrial transport would have implications for synapse density, plasticity, and transmission; furthermore, defects in mitochondrial transport have been linked to neurodegeneration (Sheng and Cai 2012). Expression of mature microRNA was beyond the scope of this study, but DE stem loop sequences were identified as were 14 novel lncRNAs (supplementary Tables 3 and 4), indicating that there is a role for non-coding RNAs in cerebral cortex maturation and aging, as previously described (Qureshi and Mehler 2011).

Although the overall results show limited changes in protein-coding genes, our study revealed that differential expression of non-coding genes and individual transcripts were prevalent: 1.3 % of all non-coding genes expressed were DE compared to 0.13 % of protein-coding genes, though non-coding genes were more DE between 6- and 12-month-old rats. This highlights the importance of expanding transcriptional analyses of aging to other levels of genomic information beyond protein-coding genes. Multiple snoRNAs, a type of non-coding RNA, were found to be DE in this study. snoRNAs generally guide the modification of ribosomal RNA, though a previous study has shown that snoRNAs can regulate alternate splicing of the serotonin receptor 2C mRNA (Kishore and Stamm 2006). High numbers of snoRNAs were down-regulated between 6- and 12-month-olds; this comparison also shows the greatest number of spliced transcripts, suggesting that non-coding RNAs are involved in regulation of splicing activity during maturation and aging.

Many non-coding RNAs also show a quadratic pattern over the three age groups and the similarity of the pattern to the protein coding genes may suggest that changes occurring with age are regulated, rather than a result of damage—as is the traditional view of aging. Closer observation of the patterns of expression in the pair-wise comparisons shows that between 12- to 28-month-olds, all non-coding genes were up-regulated and the majority of protein-coding genes were down-regulated; a similar pattern is observed in the other pair-wise comparisons. These data suggest that there is a subtle regulation of the non-coding RNA network that may affect protein-coding gene expression, splicing, promoter selection, and coding sequence output with age. Unravelling these dynamic changes in transcriptional networks with age that appear to involve multiple genome levels warrant further investigation.

Clearly transcriptome changes with age extend beyond protein-coding genes and their regulation may involve players in the dark matter of the genome. Our results suggest that changes in non-coding RNA, splice variant usage, and expression may be part of a tightly controlled regulatory non-coding RNA network that changes with age. This may be affecting the response to immune challenge, decreasing lipid clearance and altering neurotransmitter levels, providing a hypothesis and candidates for further studies. Lastly, our work demonstrates the use of RNA-seq to obtain a more detailed picture of transcriptional changes with age which we believe will be necessary to unravel the mechanisms of brain aging.

## Electronic supplementary material

Below is the link to the electronic supplementary material.

ESM 1 (XLS 35 kb)

ESM 2 (XLS 57 kb)

ESM 3 (XLS 71 kb)

ESM 4 (XLS 34 kb)

ESM 5 (XLS 57 kb)

ESM 6 (XLS 48 kb)
